# Systematic design and optimisation of layer-by-layer nanocomposite coatings on three-dimensional porous scaffolds for enhanced mechanical performance in bone tissue engineering applications

**DOI:** 10.1039/d5ra09314g

**Published:** 2026-03-04

**Authors:** MohammadAli Sahebalzamani, Helen O. McCarthy, Tanya J. Levingstone, Nicholas J. Dunne

**Affiliations:** a School of Mechanical and Manufacturing Engineering, Dublin City University Dublin 9 Ireland nicholas.dunne@dcu.ie +353 1 7005712; b Centre for Medical Engineering Research, Dublin City University Dublin 9 Ireland; c Advanced Manufacturing Research Centre (I-Form), School of Mechanical and Manufacturing Engineering, Dublin City University Dublin 9 Ireland; d Biodesign Europe, Dublin City University Dublin 9 Ireland; e Advanced Processing Technology Research Centre (APT), Dublin City University Dublin 9 Ireland; f Research Centre for Medical Devices (CÚRAM), Biomedical Sciences, University of Galway H91 W2TY Galway Ireland; g School of Pharmacy, Queen's University Belfast Belfast BT9 7BL UK; h Department of Mechanical and Manufacturing Engineering, School of Engineering, Trinity College Dublin Dublin 2 Ireland; i Advanced Materials and Bioengineering Research Centre (AMBER), Trinity College Dublin Dublin 2 Ireland; j Trinity Centre for Biomedical Engineering, Trinity Biomedical Sciences Institute, Trinity College Dublin Dublin 2 Ireland

## Abstract

Structural integrity and interconnected porosity are essential design criteria for bone tissue scaffolds. This study presents the optimisation of electrostatic layer-by-layer (LbL) assembly conditions for nanocomposite multilayer coatings on highly porous polyurethane scaffold templates, relevant to bone scaffold applications. Mechanically robust LbL coatings were applied to enhance the balance between scaffold porosity and mechanical strength. A design of experiments (DoE) approach using response surface methodology (RSM) was conducted in two stages to systematically optimise both LbL process parameters and nanocomposite composition. The optimised scaffolds exhibited a 260-fold increase in elastic modulus, from 0.09 MPa (uncoated) to 23.55 MPa, while maintaining a highly interconnected pore structure with 78.2% ± 1.24 porosity. These results establish a systematic framework for tuning LbL assembly on porous scaffolds towards bone tissue engineering.

## Abstract 2

Systematic optimisation of composition and process parameters for Layer-by-Layer assembly deposition of nanocomposite films on porous templates to identify variables governing multilayer formation, mechanical reinforcement, and porosity.

## Graphical abstract

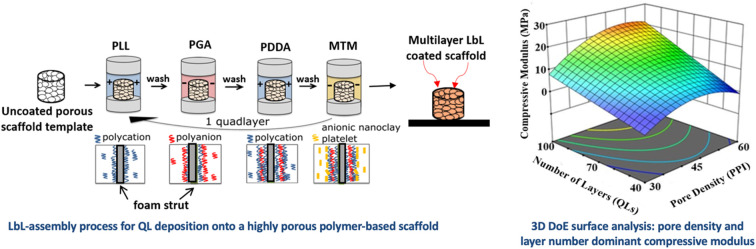

## 1 Introduction

The increasing prevalence of age-related bone disorders and the higher incidence of injuries in elderly populations have led to a growing demand for bone graft materials in orthopaedic surgery.^1–3^ Despite significant progress in synthetic graft fabrication, challenges remain in replicating native osteogenesis, simplifying manufacturing processes, and achieving an optimal balance between mechanical strength and scaffold porosity, which are factors critical for clinical translation.^4–6^ An effective synthetic bone graft must provide mechanical integrity to support load transfer and promote osteogenesis, while maintaining a highly interconnected porous structure that facilitates cell infiltration and nutrient diffusion.^7^ However, increasing porosity often reduces mechanical properties, posing a major design challenge for functional bone scaffolds.^8^

A promising approach to address this trade-off is the deposition of nanocomposite coatings onto porous polymer-based scaffolds using electrostatic layer-by-layer (LbL) assembly.^9,10^ LbL assembly is a thin-film fabrication method based on the sequential deposition of oppositely charged species, such as polyelectrolytes or micro/nanoparticles, onto a charged substrate.^11,12^ This bottom-up approach allows precise control over film thickness, composition and architecture, enabling the tuning of mechanical and physicochemical properties.^13–15^ While LbL assembly is well established on planar substrates, studies focused on using it to reinforce three-dimensional (3D) porous structures remain limited.^16^ Our recent review has highlighted its potential for producing nanocomposite-coated porous templates with tuneable properties suited to bone tissue engineering applications.^9^

Incorporating rigid nanofillers such as carbon nanotubes, carbon nanofibers, synthetic oxide nanoparticles, or nanoclays into LbL films can markedly enhance their mechanical performance.^17–19^ Montmorillonite (MTM), in particular, is a biocompatible and low-cost nanoclay with a high in-plane elastic modulus (270 GPa) that has proven effective as a reinforcing component in polymer films on planar substrates.^19,20^ Although LbL assembly is often applied to planar substrates, it can be adapted to create conformal coatings on complex 3D geometries.^21^ The selection of porous materials with appropriate interconnectivity and morphology is critical in bone scaffold design.^22,23^ The mechanical performance of scaffold-type materials depends on factors such as porosity, pore shape, and base material.^24,25^ Adding nanoscale fillers reinforces polymer-based scaffolds through imparting interactions at the filler–matrix interface, improving stiffness and strength without severely compromising porosity.^26^

Open-cell polyurethane (PU) foam has potential due to its biocompatibility, tuneable porosity, and architectural similarity to cancellous bone, making it a potential option as a bone graft.^9,27,28^ The modular nature of LbL assembly facilitates accurate control over the number of deposited layers, enabling systematic tailoring of cellular mechanics while retaining high porosity, suggesting their potential efficacy in the development of tissue-engineered bone scaffolds.^9,29^ Previous work has shown that polymer-nanoclay LbL coatings significantly improve the mechanical and physical properties of 3D porous polymer-based scaffolds.^14,15,30^ For example, Ziminska *et al.* demonstrated that P-based coated with 40 quadlayers (QL) of polyethyleneimine (PEI), polyacrylic acid (PAA), PEI and MTM increased in mass, coating thickness and bulk elastic modulus (1.73 MPa *versus* 0.07 MPa for uncoated scaffolds).^14^ Similarly, PEI/PAA/PEI/MTM QL coatings yielded up to a 25-fold increase in strength while maintaining (88%) porosity.^30^ McFerran *et al.* used PEI, poly(diallydimethylammonium chloride) (PDDA), chitosan (CHI) and MTM to achieve tuneable mechanical properties while maintaining 95% porosity after 15 QL and 88% porosity after 60 QL.^31^ Prior work by our group reported a 40-fold improvement in mechanical performance through 40 QL deposition of poly-l-lysine monohydrochloride (PLL), poly-l-glutamic acid (PGA), PDDA and MTM on PU-based scaffolds while maintaining a porosity of 86% porosity and interconnectivity.^16^ These findings confirm that LbL assembly can improve scaffold mechanical properties without compromising their essential porous structure for bone tissue engineering applications.^31^ Comprehensive understanding of the factors influencing these coatings is therefore essential for process optimisation.

Beyond mechanical reinforcement, LbL coatings can also modify the surface to enhance biological response or facilitate chemical functionalisation with drugs or biomolecules.^11^ Mertz *et al.* developed nanoscale barrier films using alternate deposition of PDDA/polystyrene sulfonate (PSS) and PLL/hyaluronic acid (HA).^32^ Kim *et al.* produced nanocomposite coatings from hydroxyapatite and collagen, improving cell adhesion and proliferation on a biodegradable polymer-based scaffold.^33^ Lee *et al.* deposited PDDA/MTM coatings on inverted colloidal crystal scaffolds to increase surface roughness and stiffness, which promoted epithelial cell adhesion and migration.^34^ Andres *et al.* similarly used polyvinyl alcohol (PVA) and MTM nanoclay to deposit multilayer coatings onto inverted colloidal crystal scaffolds with interconnected and tuneable pore architecture.^35^ While these studies highlight the versatility of LbL coatings in tuning scaffold properties, a systematic optimisation of the LbL assembly process itself remains underexplored.^36^

Many commonly used LbL components, including PEI, PAA, PLL, and PGA, are weak polyelectrolytes whose charge density depends on pH.^37,38^ Thus, coating properties can be tuned by adjusting solution pH and concentration, which modify polymer conformation and diffusion within the multilayer structure.^39–41^ Other factors, such as immersion time, drying conditions, molecular weight, and temperature, also influence film properties.^9,29,42^ The internal geometry of the scaffold and the number of deposited QLs further affect the coating's overall performance.^9,11^ However, systematic studies evaluating how these parameters collectively influence porosity, mechanical strength, and coating uniformity, particularly for PLL/PGA/PDDA/MTM systems, are scarce.

Given the complex interplay between process parameters, a design of experiments (DoE) approach provides an efficient, statistically guided framework for optimisation.^43^ DoE identifies relationships between processing variables and material responses, reducing experimental workload while revealing factor interactions.^43–45^ In this study, LbL assembly conditions and material parameters were systematically optimised using a DoE approach to enhance the mechanical and structural properties of nanocomposite-coated polymer-based scaffolds. The goal was to establish relationships between key process variables and scaffold performance, enabling the fabrication of tuneable, mechanically robust constructs for bone tissue engineering applications.

## 2 Materials & methods

### 2.1 Materials system

Nanocomposite-biopolymer films were deposited using an LbL assembly process. The polyelectrolyte solutions consisted of PLL, PGA, PDDA (Sigma Aldrich, Ireland), and MTM (BYK, UK). A multilayered LbL coating was established by alternating deposition of 1 wt% PLL (Mw: 182.65 g mol^−1^, positive), 1 wt% PGA (Mw: 147.13 g mol^−1^, negative), 1 wt% PDDA (Mw: 400 kDa, 20 wt% in H_2_O, positive), and 0.5 wt% MTM (negative) solutions onto open-cell polyurethane (PU) foam-based scaffolds (12 mm diameter, 10 mm height; EasyFoam Ltd., UK) with pore densities of 30, 45, or 60 pores per inch (PPI). All aqueous solutions were diluted in deionised water (resistivity ≥18.2 MΩ) under vigorous stirring for 24 h, with the pH adjusted as previously described.^16^

### 2.2 LbL assembly process

**Fig. 1:**
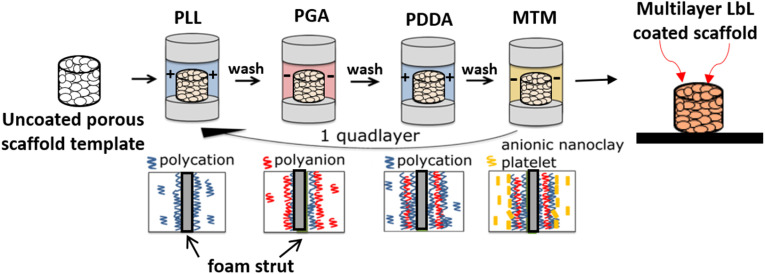
Schematic of the LbL-assembly process for QL deposition of PLL/PGA/PDDA/MTM onto a highly porous and interconnected polymer-based scaffold using a customised deposition chamber.

Multilayer coatings were deposited in a custom-designed LbL chamber under dynamic conditions involving perfusion (12 mL min^−1^, 10 rpm) and cyclic compressive loading (5% strain, 1 Hz), as optimised previously.^16^ PU scaffolds were exposed to alternating solutions of PLL (+), PGA (−), PDDA (+), and MTM (−), creating a nanocomposite QL thin film (Fig. 1). Each solution introduction was followed by three DI water rinses (≥30 s contact time each). Every 10 QLs, the scaffold was rinsed three times with DI water and air dried for 24 h (approximately 23 °C, 30% humidity). The full sequence was repeated as necessary to achieve the target QLs. A two-phase DoE approach optimised both material system and process conditions, specifically polymer/nanoclay concentration, pH, contact time, number of layers, drying frequency, and scaffold pore density.

### 2.3 Experimental design

DoE study 1 focused on the optimisation of nanocomposite LbL material conditions using a two-level factorial design (1/4 fractional factorial, Design-Expert V5 Software, Stat-Ease Inc., USA), which analysed the main effects and interactions of six variables (concentration and pH for each material, Table 1). The two-level factorial design is the most common method for analysing the response variable when the main effect of each variable and the interaction between two or more variables on the output are considered.^43^ Literature informed the selection of the input factor.^9,16,30^ The design comprised 20 experimental runs with randomised order (Table 2) and measured mass, porosity, strut thickness, and compressive elastic modulus, using PU scaffolds at 30 PPI as templates. Solution contact time was kept constant (30 s); after every 10 QLs, samples were rinsed three times and dried in a desiccator for 24 h. Thirty QLs were achieved by repeating the coating cycle three times.

**Table 1:** Experimental parameter ranges for DoE study 1. Parameters A–D: pH of PLL, PGA, PDDA, MTM; parameters E and F: concentrations of polymers and MTM. Lower and higher input values for each parameter are indicated

Factor	Parameter	Units	Lower level	Upper level
A	pH of PLL	—	3	7
B	pH of PGA	—	7	11
C	pH of PDDA	—	4	7
D	pH of MTM	—	6	11
E	Polymer concentration	wt%	1	5
F	Nanoclay concentration	wt%	0.05	1.5

**Table 2:** Experimental runs in DoE study 1 with factors A–F corresponding to material system conditions

Run	Factor A: PLL pH	Factor B: PGA pH	Factor C: PDDA pH	Factor D: MTM pH	Factor E: polymers concentration (wt%)	Factor F: MTM nanoclay concentration (wt%)
1	7	7	4	11	5	1.5
2	7	7	7	6	1	1.5
3	7	11	7	6	5	0.5
4	7	11	7	11	5	1.5
5	3	7	4	11	5	0.5
6	3	9	7	11	5	0.5
7	5	9	5.5	8.5	3	1
8	5	9	5.5	8.5	3	1
9	5	11	5.5	8.5	3	1
10	7	11	4	11	1	0.5
11	3	7	7	6	1	0.5
12	3	7	7	6	5	1.5
13	3	7	4	11	1	1.5
14	3	7	4	6	1	0.5
15	7	11	7	11	1	0.5
16	3	7	7	11	1	1.5
17	7	7	4	6	5	0.5
18	7	11	4	6	1	1.5
19	3	11	4	6	5	1.5
20	5	9	5.5	8.5	3	1

DoE study 2 investigated and optimised LbL process conditions using a Box–Behnken design (BBD, Design-Expert software), with four key factors (pore density, number of layers, drying frequency, contact time) at three levels (Table 3), determined from literature.^9,15,16,46^ The BBD included 28 experimental runs with randomised order (Table 4) and measured mass, porosity, coating thickness, and compressive elastic modulus (*n* = 5), with replication points used for independent error estimation and model curvature assessment. Coating assembly parameters were fixed at optimal values drawn from DoE study 1: pH values (PLL 3.5, PGA 10, PDDA 4.5, MTM 9.5), polymer concentration (1 wt%), MTM concentration (0.5 wt%), pore density (45 PPI), 100 layers, drying every 10 QLs, and 30 s contact time.

**Table 3:** Parameter ranges (A–D: pore density, number of layers, drying frequency, contact time) for DoE study 2. Lower and higher input values for each parameter are indicated

Factor	Parameter	Units	Lower level	Upper level
A	Pore density of scaffolds	PPI	30	60
B	Number of layers	QLs	40	100
C	Frequency of drying	QLs	10	20
D	Contact time	s	30	60

**Table 4:** Experimental runs in DoE study 2 with factors A–D for LbL assembly process conditions

Run	Factor A: pore density (PPI)	Factor B: number of layers (QLs)	Factor C: drying steps (QLs)	Factor D: coating time (s)
1	30	70	20	45
2	30	40	15	45
3	45	70	15	45
4	45	40	20	45
5	45	100	15	60
6	45	40	10	45
7	60	70	20	45
8	60	70	15	60
9	45	70	15	45
10	30	100	15	45
11	60	40	15	45
12	45	70	15	45
13	45	70	10	30
14	45	70	15	45
15	45	100	20	45
16	45	40	15	60
17	60	70	15	30
18	45	70	10	60
19	45	40	15	30
20	30	70	10	45
21	45	100	10	45
22	60	70	10	45
23	45	100	15	30
24	30	70	15	60
25	45	70	20	30
26	30	70	15	30
27	45	70	20	60
28	60	100	15	45

### 2.4 DoE optimisation

Numerical optimisation was conducted using Design-Expert software to identify optimal settings for multilayer coatings. Desirability functions maximised the elastic modulus and porosity while minimising thickness and mass. Those criteria defined input parameters and responses (Tables 5 and 6 for DoE study 1 and DoE study 2, respectively).

**Table 5:** DoE study 1 optimisation process for determining the optimal material system parameters. Parameters A–F represent all input variables, along with four output variables of the LbL-assembled coated scaffolds. The optimisation goals, as well as the lower and upper limits for each input and output parameter, are also indicated

Factors	Goal	Lower level	Upper level
A: pH PLL	In range	3	7
B: pH PGA	In range	7	11
C: pH PDDA	In range	4	7
D: pH MTM	In range	6	11
E: polymer concentration (wt%)	In range	0.5	5
F: clay concentration (wt%)	In range	0.5	1.5
Mass (mg)	Minimise	39.3	63.8
Coating thickness (µm)	Minimise	133.46	189.62
Compression modulus (MPa)	Maximise	0.90	4.20
Porosity (%)	Maximise	90.13	96.30

**Table 6:** DoE study 2 optimisation process for determining the optimal LbL assembly process parameters. Parameters A–F represent all input variables, along with four output variables of the LbL-assembled coated scaffolds. The optimisation goals, as well as the lower and upper limits for each input and output parameter, are also indicated

Factors	Goal	Lower level	Upper level
A: pore density of scaffolds	In range	30	60
B: number of layers	In range	40	100
C: frequency of drying	In range	10	20
D: contact time	In range	30	60
Mass (g)	Minimise	0.10	0.22
Coating thickness (µm)	Minimise	5.7	19.1
Compression modulus (MPa)	Maximise	0.64	25.2
Porosity (%)	Maximise	75.5	89.1

LabL-coated scaffolds were fabricated under optimised conditions described in DoE study 1 and DoE study 2, and responses were measured to validate models and optimisation outcomes (*n* = 5). Additional characterisation included energy-dispersive X-ray spectroscopy (EDX), Fourier transform infrared (FTIR) spectroscopy, X-ray diffraction (XRD), contact angle analysis, and mechanical testing.

### 2.5 Physico-mechanical characterisation

#### 2.5.1 Gravimetric analysis

Mass of coated and uncoated scaffolds (*n* = 5) was measured using an analytical balance (0.0001 g resolution) after each drying cycle. Mass per QL was calculated by dividing the coating mass by the number of QL deposited.

#### 2.5.2 Microscopy

SEM characterised scaffold topography of coated and uncoated scaffolds (*n* = 5, Zeiss EVO LS15, Germany, 18 kV). Specimens were fixed onto an aluminium disc and gold sputter-coated (Edwards, West Sussex) at 60 mA for 80 s to minimise charging.

#### 2.5.3 Coating thickness

Cross-sections of coated scaffolds were assessed using SEM to determine the average coating thickness at 10 data points from five images per sample.

#### 2.5.4 Mechanical properties

Uniaxial compression testing assessed scaffold modulus (BioTester, Univert, Canada, 100 N load cell, *n* = 5); compression to 0.6 mm, speed 2 mm min^−1^, preload 0.03 N. Specimen dimensions were recorded using digital Vernier callipers and averaged from three measurements. The compressive modulus was calculated as the slope of the linear region of the stress–strain curve. Uncoated PU scaffolds (30, 45, 60 PPI) provided baseline controls.

#### 2.5.5 Porosity

Porosity was determined gravimetrically (*n* = 5), measuring mass before and after water immersion.^16^ The scaffold volume was calculated using a digital Vernier calliper; the density (*ρ*_s_) and porosity were determined using the following standard formulas. Theoretical density (*ρ*_m_) for the uncoated PU scaffold was denoted as 1.420 g cm^−3^.^47^ Percentage porosity was assessed using a density kit (LabQuip, Ireland). The density (*ρ*_s_) and percentage porosity of each scaffold was determined using the following equations:
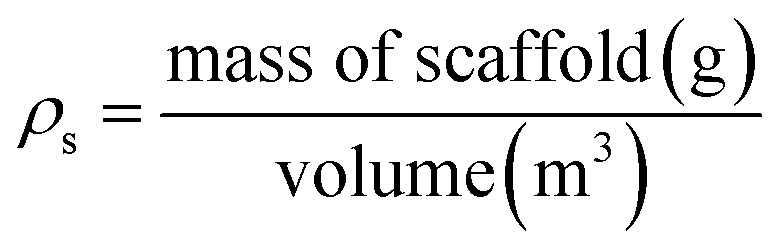

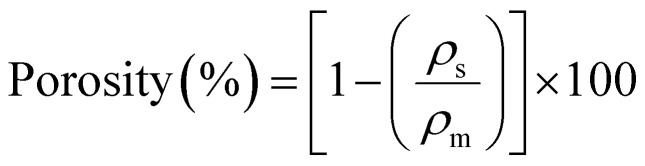
where *ρ*_s_ is the measure scaffold density and *ρ*_m_ is the solid density of the scaffold material, *i.e.*, free from pores or air bubbles. Controls included uncoated scaffolds.

#### 2.5.6 Surface morphology and chemical characterisation

Surface morphology and elemental composition were assessed *via* point mode on five data regions scanning at different point locations per sample (*n* = 5) using SEM coupled with energy-dispersive X-ray analysis (SEM-EDX) (Zeiss EVO LS15, Germany). The resulting spectra provided detailed elemental profiles.

Chemical composition of the LbL coating was determined by Attenuated total reflectance (ATR) Fourier-transform infrared (FTIR) spectroscopy (PerkinElmer Spectrum 100). Spectra (*n* = 5) within the range of 500–4000 cm^−1^ were obtained with an average of 64 scans per spectrum. The FTIR transmission was normalised for peak comparison. Functional groups were identified based on absorbance ranges corresponding to analogous organic and inorganic compounds.

X-ray Diffraction (XRD, Bruker D8, Germany) assessed MTM presence (*n* = 5). Characterisation was conducted at 30 kV and 10 mA, with a scan speed of 2 s per scan using Cu-Kα radiation between a 2*θ* of 20° and 80°. The resultant spectra were compared using the JCPDS database and the X'Pert High Score Plus software (X'Pert, Netherlands).

#### 2.5.7 Wettability of coating

Wettability (*n* = 5) was quantified using contact angle analysis using the sessile drop method. A 5 µL DI water droplet was placed on each scaffold, and angles were measured with an FTÅ200 Dynamic Contact Angle Analyser (First Ten Angstroms, USA).

#### 2.5.8 Statistical analysis

Data were presented as mean ± standard deviation (*n* = 5). Statistical analyses used the DoE software, with significance assessed by regression, analysis of variance method (ANOVA), effect plots, and appropriate tests (*p*-value < 0.05). For DoE study 1, half-normal probability and effect plots determined significant factors. Data transformation was optimised using the square root based on Box–Cox and *λ* value analysis. For DoE study 2, regression models and effect plots quantified factor contributions.^44^ Statistical significance and model adequacy applied sequential *F*-tests and lack-of-fit tests (*p*-value < 0.05).

## 3 Results

### 3.1 Optimisation of LbL nanocomposite coating parameters: DoE study 1

#### 3.1.1 DoE study 1 analysis approach

A two-level, 1/4 fractional factorial DoE (six factors) was used to identify principal effects and interactions influencing coating properties (Table 7).

**Table 7:** Experimental runs results for the DoE study 1 based on the four output variables of the LbL-assembled coated scaffolds

Run	Mass (mg)	Strut coating thickness (µm)	Compressive modulus (MPa)	Porosity (%)
1	39.40	138.46	1.74	95.91
2	46.10	156.13	2.15	96.10
3	39.30	158.89	0.90	92.33
4	48.20	148.71	2.80	95.01
5	48.60	189.62	2.65	92.51
6	49.80	145.33	3.80	95.69
7	39.50	140.09	1.95	91.79
8	52.90	151.13	2.90	95.25
9	39.90	142.89	1.70	95.53
10	60.50	148.71	4.20	95.86
11	45.10	169.62	3.35	92.89
12	54.60	135.76	3.85	95.36
13	63.80	133.46	4.10	90.13
14	44.40	141.11	2.15	96.19
15	51.70	141.69	3.01	91.23
16	43.40	135.25	3.60	96.30
17	47.20	136.10	2.40	94.30
18	59.30	141.69	3.35	92.77
19	43.50	138.75	2.45	95.14
20	44.80	139.23	2.25	93.66

#### 3.1.2 Mass increase of deposited LbL coating

**Fig. 2:**
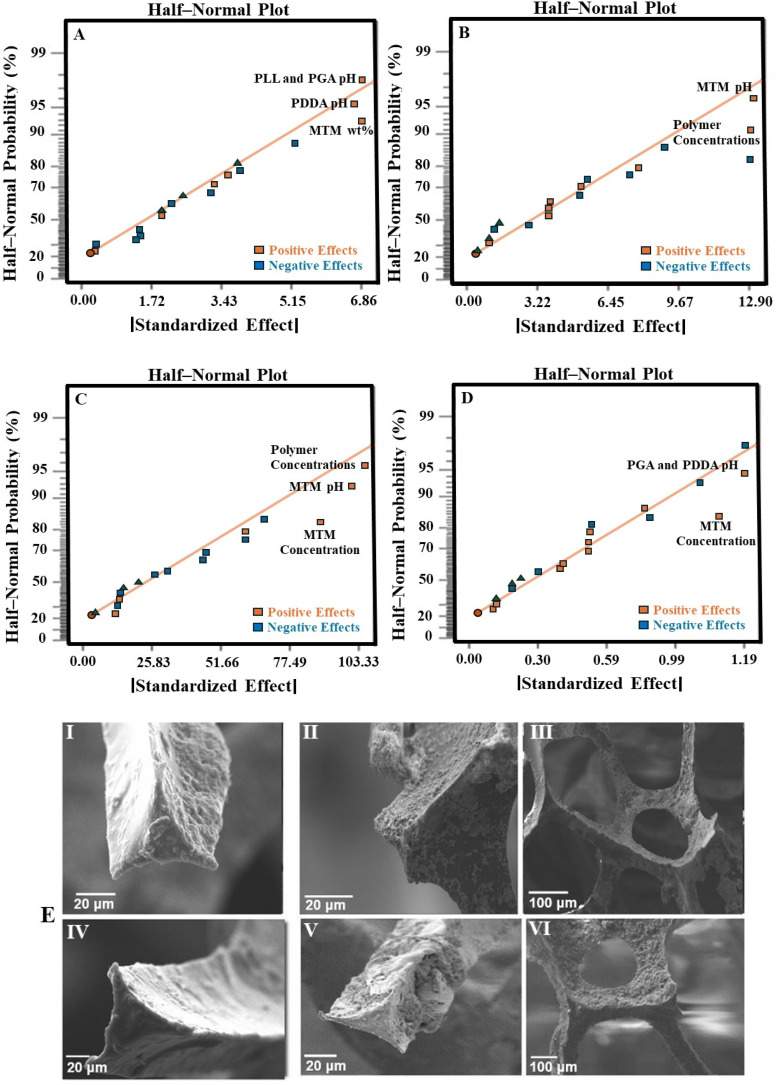
The half-normal probability plots and effect plots (A–D) were used to determine the main factors affecting each of the measured properties. Half-normal plot of the standardised effects for the influence of inputs on: (A) the mass increase of deposited nanocomposite coating, (B) LbL assembled coating thickness, (C) compressive modulus of LbL-coated scaffolds and (D) the porosity of LbL-coated scaffolds. (E) SEM images showing the coated surfaces of the scaffold following the LbL assembly process using low/high values for different factors. (I) High polymer concentrations (Run: 6). (II) Low MTM pH and low MTM concentration (Run: 11). (III) High polymer pH and low MTM pH (Run: 12). (IV) Low polymer concentrations (Run: 15). (V) High MTM concentrations (Run: 16). (VI) Low polymer and MTM concentrations (Run: 14).

The mass of the uncoated scaffold (30 PPI) was 36.1 ± 0.5 mg. LbL coating (30 QLs) resulted in scaffolds with masses ranging from 39.4 ± 0.3 mg to 63.8 ± 0.7 mg. The MTM nanoclay concentration, PDDA pH, and the interaction between PLL and PGA pH had significant effects on the deposited mass (*p*-value < 0.0001, *R*^2^ = 0.8609, Fig. 2A). The MTM concentration dominated the response; other factors were not significant.

#### 3.1.3 Multilayer strut thickness

SEM imaging showed the uncoated scaffold had an average strut thickness of 125 ± 2.2 µm. After coating with 30 QLs, thicknesses ranged from 135.25 µm to 189.62 µm. MTM pH and polymer concentrations (*p*-value < 0.0001, *R*^2^ = 0.8410) significantly increased coating thickness (Fig. 2B). Other factors were insignificant.

#### 3.1.4 Mechanical properties

Uncoated scaffolds exhibited a compressive elastic modulus of 0.09 ± 0.01 MPa. LbL-coated scaffolds had moduli from 0.90 MPa to 4.20 MPa. Polymer concentrations, MTM pH, and nanoclay concentration significantly affected mechanical properties (*p*-value < 0.0001, *R*^2^ = 0.8920, Fig. 2C).

#### 3.1.5 Porosity

Uncoated scaffolds had 97.4 ± 1.1% porosity; coated scaffolds (30 QLs) ranged from 90.13% to 96.30%. MTM concentration and the interaction between PDDA pH and PGA pH significantly reduced porosity when nanoclay concentration increased (*p*-value < 0.0001, *R*^2^ = 0.8110, Fig. 2D). High PDDA pH and low PGA pH achieved the highest porosity, likely due to their effect on polymer charge density and LbL uniformity.^31^ Other factors were not significant.

#### 3.1.6 Optimisation of LbL assembly material parameters

Parameter selection for optimisation yielded a desirability index of 1. Optimal concentrations were 1 wt% for polymers and 0.5 wt% for nanoclay. Optimum pH values were PLL (3.5), PGA,^10^ PDDA (4.5), and MTM (9.5), supporting maximum polyelectrolyte charge density (Table 8). *T*-test analysis between predicted and experimental results for optimised scaffolds showed a non-significant difference (*p*-value = 0.1, <4% difference, Table 9). The compressive modulus improved from 1.90 MPa (unoptimised) to 3.80 MPa, and porosity from 90.48% to 91.85%. Thus, optimised coating conditions enabled targeted enhancement of scaffold properties for bone tissue engineering applications.

**Table 8:** Optimal conditions, materials parameters for the deposition of nanocomposite coatings onto porous scaffolds using LbL assembly based on the DoE study 1 optimisation analysis

Optimal materials	pH PLL	pH PGA	pH PDDA	pH MTM	Polymers concentration	Clay concentration
Parameters	3.5	10	4.5	9.5	1 wt%	0.5 wt%

**Table 9:** Validation and comparison of the difference between predicted responses from DoE optimisation and actual values from experimental work, using a composition of the optimal outputs (*n* = 5)

Responses	Actual responses (mean ± SD)	Predicated responses (mean)	Error (%)
Mass (mg)	50.10 ± 1.4	49.05	2.1
Strut thickness (µm)	153.25 ± 1.7	147.55	3.7
Compressive elastic modulus (MPa)	3.80 ± 0.20	3.65	3.1
Porosity (%)	91.85 ± 0.91	92.50	1.3

### 3.2 Optimisation of LbL assembly process parameters: DoE study 2

In DoE study 2, the scaffolds were coated with PLL/PGA/PDDA/MTM QLs using the optimal parameters from DoE study 1. Mass, thickness, porosity, and compressive modulus were characterised for various numbers of QLs and scaffold pore densities (Table 10). Baseline values for uncoated scaffolds of 30, 45, and 60 PPI are provided in Table 11.

**Table 10:** Experimental runs results for the DoE study 2 based on the four output variables of the LbL-assembled coated scaffolds

Run	Factor A: pore density (PPI)	Factor B: number of layers (QLs)	Factor C: drying steps (QLs)	Factor D: coating time (s)	Mass (g)	Coating thickness (µm)	Compressive modulus (MPa)	Porosity (%)
1	30	70	20	45	0.157	8.9	1.85	83.60
2	30	40	15	45	0.108	5.8	0.644	85.80
3	45	70	15	45	0.200	12.6	13.01	84.80
4	45	40	20	45	0.158	6.3	3.86	84.50
5	45	100	15	60	0.216	19.1	22.5	80.20
6	45	40	10	45	0.146	5.7	3.88	87.30
7	60	70	20	45	0.175	12.9	10.21	80.20
8	60	70	15	60	0.196	12.3	9.85	78.50
9	45	70	15	45	0.189	12.5	13.15	85.10
10	30	100	15	45	0.161	13.1	5.56	83.00
11	60	40	15	45	0.112	6.1	2.45	89.01
12	45	70	15	45	0.201	12.3	13.14	84.90
13	45	70	10	30	0.191	12.1	13.21	85.50
14	45	70	15	45	0.198	12.4	12.92	85.10
15	45	100	20	45	0.207	18.2	21.35	80.00
16	45	40	15	60	0.163	6.7	3.09	86.40
17	60	70	15	30	0.185	11.8	10.41	81.20
18	45	70	10	60	0.225	13.6	12.46	83.10
19	45	40	15	30	0.152	5.9	4.35	86.90
20	30	70	10	45	0.135	7.6	2.38	86.10
21	45	100	10	45	0.197	16.2	25.20	82.40
22	60	70	10	45	0.170	11.4	11.10	84.00
23	45	100	15	30	0.205	16.8	23.51	81.00
24	30	70	15	60	0.161	8.4	2.03	85.20
25	45	70	20	30	0.220	12.9	11.87	85.70
26	30	70	15	30	0.151	7.1	2.20	84.50
27	45	70	20	60	0.238	13.3	11.79	86.40
28	60	100	15	45	0.188	17.3	18.10	79.50

**Table 11:** Properties of the different uncoated scaffold types (mean ± standard deviation (*n* = 5))

PU scaffold type	Mass	Compressive elastic modulus	Porosity
30 PPI	36.1 ± 2.3 mg	0.09 ± 0.01 MPa	97.4 ± 1.26%
45 PPI	40.4 ± 3.1 mg	0.10 ± 0.05 MPa	97.9 ± 1.43%
60 PPI	23.2 ± 1.7 mg	0.13 ± 0.01 MPa	98.8 ± 1.1%

#### 3.2.1 Mass increase of deposited LbL-assembled coating

**Fig. 3:**
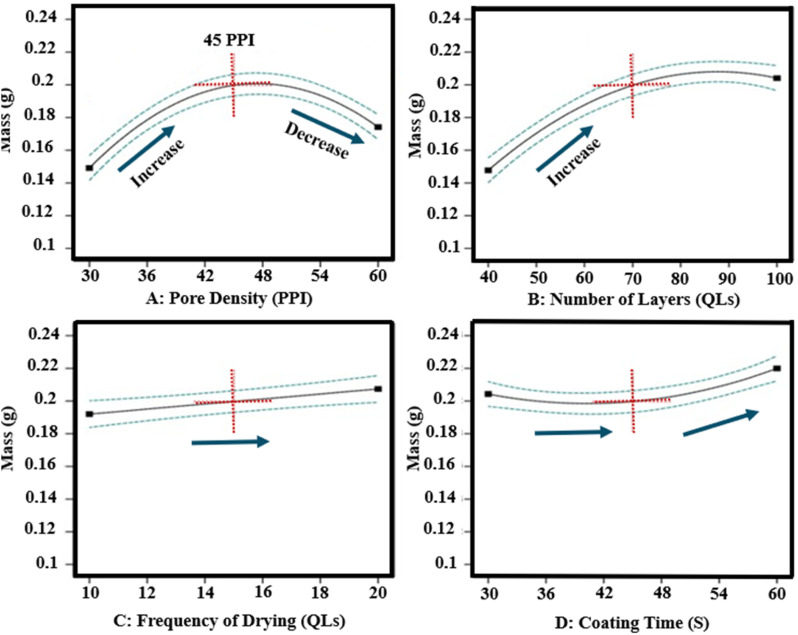
Influence of all input factors on mass response of LbL-assembled coated scaffolds according to DoE analysis.

The mass increase of LbL-coated scaffolds ranged from 0.108 g to 0.225 g. ANOVA demonstrated that scaffold pore density (Factor A) and the number of deposited layers (Factor B) had the predominant influence on mass increase, accounting for 42–49% of observed variance (*p* = 0.0097; Fig. 3), while other factors contributed 3–9%. Increasing the number of layers from 40 to 100 QLs resulted in a marked mass increase.

Among scaffold types, the 45 PPI scaffold consistently exhibited the highest mass increase. This effect can be attributed to a favourable balance between available surface area and porosity, which promotes efficient coating deposition and penetration. In contrast, the 30 PPI scaffold's larger pores provided less total surface area, limiting deposition, while the 60 PPI scaffold's smaller pores reduced effective penetrability and thus mass gain. A quadratic correlation was observed between the number of layers and mass increase, which agrees with previous investigations.^15,30^

Significant factor interactions included AB, AD (where *D* represents contact time), and ABD; all other investigated factors were found insignificant and were retained to estimate experimental error. Overall, these findings confirm that optimising pore density and layer number is essential for maximising coating deposition on 3D porous scaffolds.

#### 3.2.2 Multilayer coating thickness

**Fig. 4:**
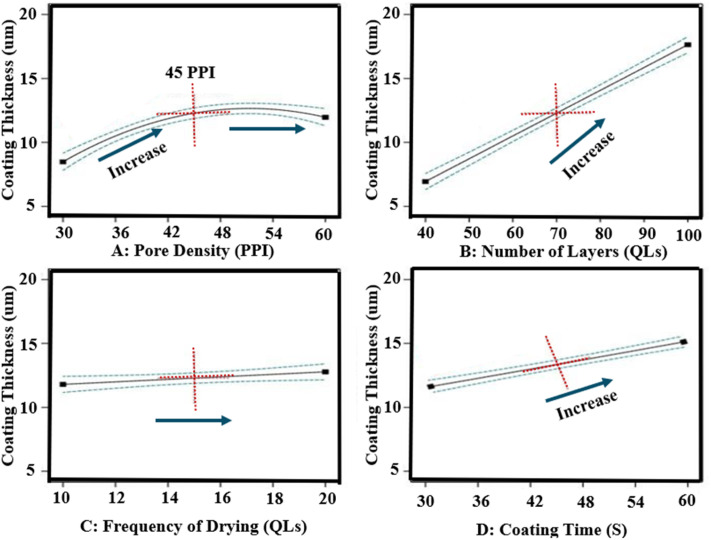
Influence of all input factors on coating thickness response of LbL-assembled coated scaffolds according to DoE analysis.

**Fig. 5:**
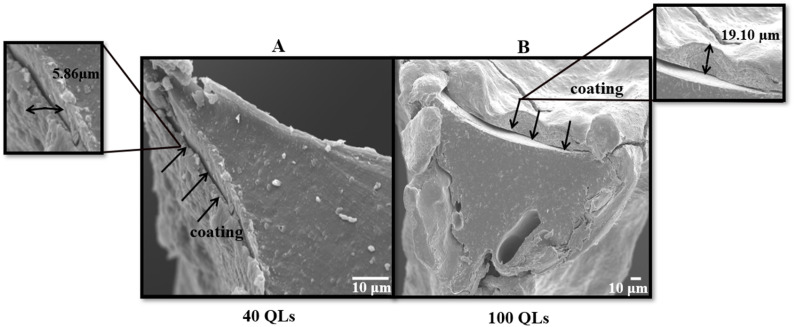
The effect of the number of layers on coating thickness. (A) SEM images for the lowest thickness LbL coating and (B) for the highest thickness LbL coating.

The thickness of LbL-assembled coatings on the scaffolds ranged from 5.7 µm to 19.1 µm. ANOVA analysis indicated a statistically significant model (*p*-value < 0.0001). The number of layers was the most influential factor, contributing more than 20% to the variation in coating thickness, as shown in the sum of squares analysis. Scaffold pore density, coating time, and drying time also had measurable effects. Increasing both the number of layers and, to a lesser degree, the coating time resulted in increased coating thickness (Fig. 4). The impact of these physical parameters is illustrated in Fig. 5, which presents SEM images of scaffolds with the minimum and maximum coating thickness observed. A linear relationship between coating thickness, number of layers, and coating time was evident, corroborating the trends described by Ziminska *et al.* and Acheson *et al.*^15,46^

#### 3.2.3 Mechanical properties of scaffolds

**Fig. 6:**
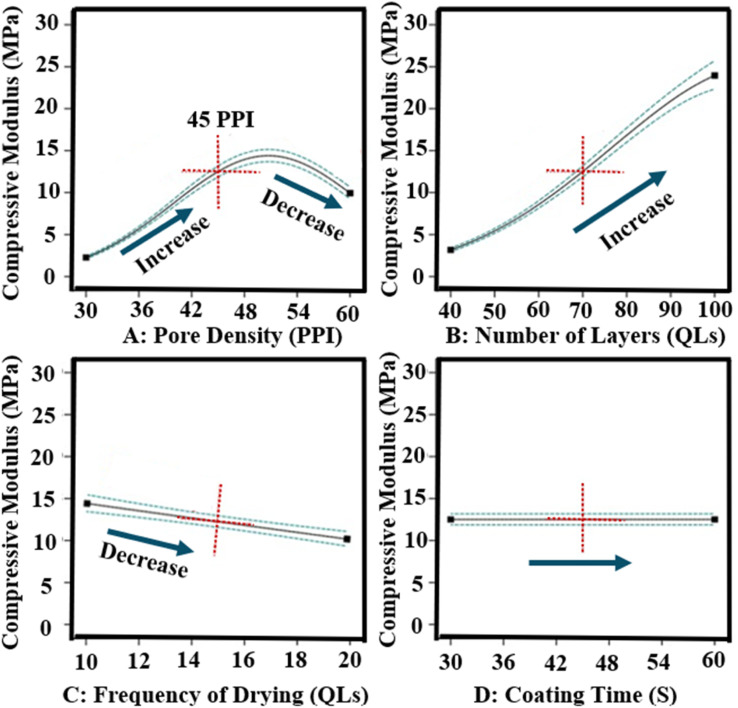
Influence of all input factors on the mechanical properties of LbL-assembled coated scaffolds according to DoE analysis.

**Fig. 7:**
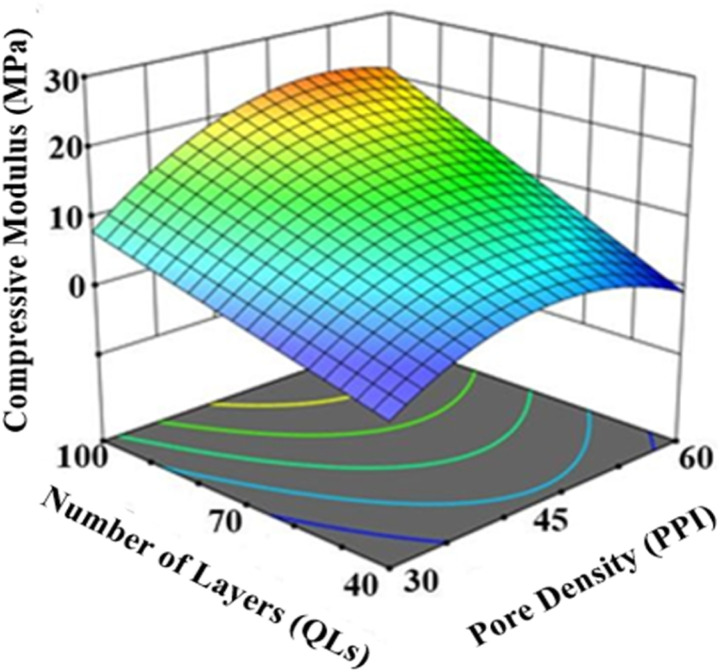
3D surface DoE analysis showed the influence of pore density and number of layers as the parameters with the greatest effect on the compressive modulus of LbL-assembled scaffolds.

The compressive modulus of LbL-coated scaffolds ranged from 0.64 MPa to 25.20 MPa. The dominant factors influencing the compressive modulus were pore density (Factor A), number of coating layers (Factor B), and their interaction (AB). The modulus trends were consistent with those observed for coating thickness and mass, as thicker coatings yielded stiffer scaffolds. ANOVA analysis of the DoE data indicated the number of layers had the largest effect, contributing over 40% to the variance (*p*-value = 0.0015). Other factors and their effects on compressive properties are summarised in Fig. 6. A decrease in compressive modulus was observed when the drying frequency (Factor C) was reduced from every 10 QLs to every 20 QLs. Pore density exhibited a quadratic relationship with compressive modulus, with 45 PPI scaffolds achieving the highest stiffness. Coating time did not significantly impact the elastic modulus. 3D surface analysis further illustrated the combined influence of pore density and number of layers, showing a strong positive correlation with compressive modulus (Fig. 7). These variations demonstrate the critical role of these parameters in optimising scaffold mechanical performance.

#### 3.2.4 Porosity

**Fig. 8:**
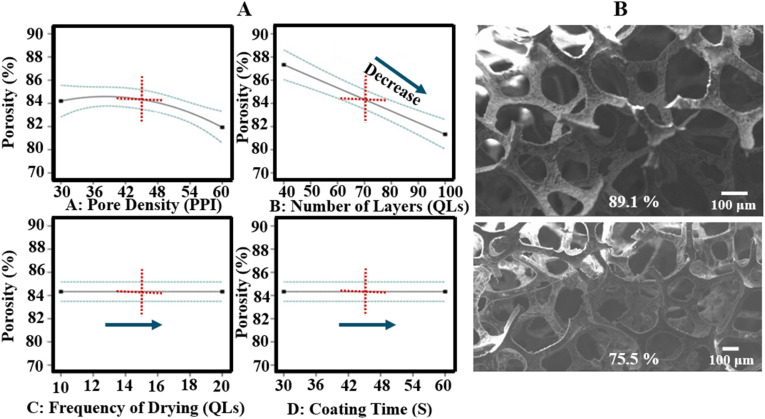
(A) Influence of all input factors on porosity of LbL-assembled scaffolds according to DoE analysis, and (B) physical data (SEM) showing the pores of the highest and lowest porosity scaffolds.

The porosity of LbL-coated scaffolds ranged from 75.5% to 89.1%. The ANOVA model for the DoE indicated that porosity was primarily influenced by the number of deposited layers, accounting for approximately 44% of the variation (*p*-value < 0.0001). This finding is consistent with the filling of pore space by the coating material, which reduces overall porosity. The number of bilayers showed an inverse relationship with porosity relative to thickness, mass, and elastic modulus. High porosity in scaffolds is desirable to provide a larger surface area for cellular attachment and facilitate cell infiltration. The decrease in porosity with increasing layers did not preclude improvements in mechanical properties, as a highly interconnected pore structure was maintained. Porosity decreased with an increase in the number of layers from 40 to 100 and with increasing scaffold pore density from 30 PPI to 60 PPI, as illustrated in Fig. 8. Coating time and drying frequency did not significantly affect porosity.

#### 3.2.5 Property optimisation of the LbL assembly processing condition

The optimisation of processing parameters aimed to balance mechanical properties, porosity, thickness, and mass to meet the design requirements for bone scaffold applications. Optimal LbL assembly conditions were identified using the DoE optimisation process, as summarised in Table 12 (desirability = 0.928). Predicted parameter values and levels necessary to achieve these properties are provided in Table 13. LbL-coated scaffolds were fabricated under these optimised conditions, and their properties were measured to validate the model predictions. A small deviation between measured and predicted values is expected, as factors identified as individually insignificant may collectively cause minor variations. *T*-test analysis indicated no significant difference between predicted and actual responses (*p*-value = 0.1), with percentage differences within 3%. These optimised parameters support the production of scaffolds that are mechanically robust and highly porous, suitable for cell infiltration in biomedical applications.^9,48^

**Table 12:** Optimal conditions of LbL process parameters for LbL-assembled coated scaffolds based on the DoE study 2 optimisation analysis

LbL process parameters	Pore density (PPI)	Number of layers (QLs)	Drying step (QLs)	Coating time (s)
Optimisation	45	100	10	30

**Table 13:** Validation and comparison of the difference between predicted responses from the DoE optimisation and actual values from experimental work, using a composition of the optimal outputs

Response	Actual responses (mean ± SD)	Predicated responses (mean)	Error (%)
Mass (g)	0.202 ± 0.03	0.199	1.50
Thickness (µm)	17.30 ± 2.04	16.84	2.7
Porosity (%)	80.66 ± 1.15	81.71	1.29
Compressive elastic modulus (MPa)	23.55 ± 3.80	24.25	3.01

### 3.3 Property optimisation of the LbL assembly materials and processing conditions

#### 3.3.1 Surface morphology and elemental analysis

**Fig. 9:**
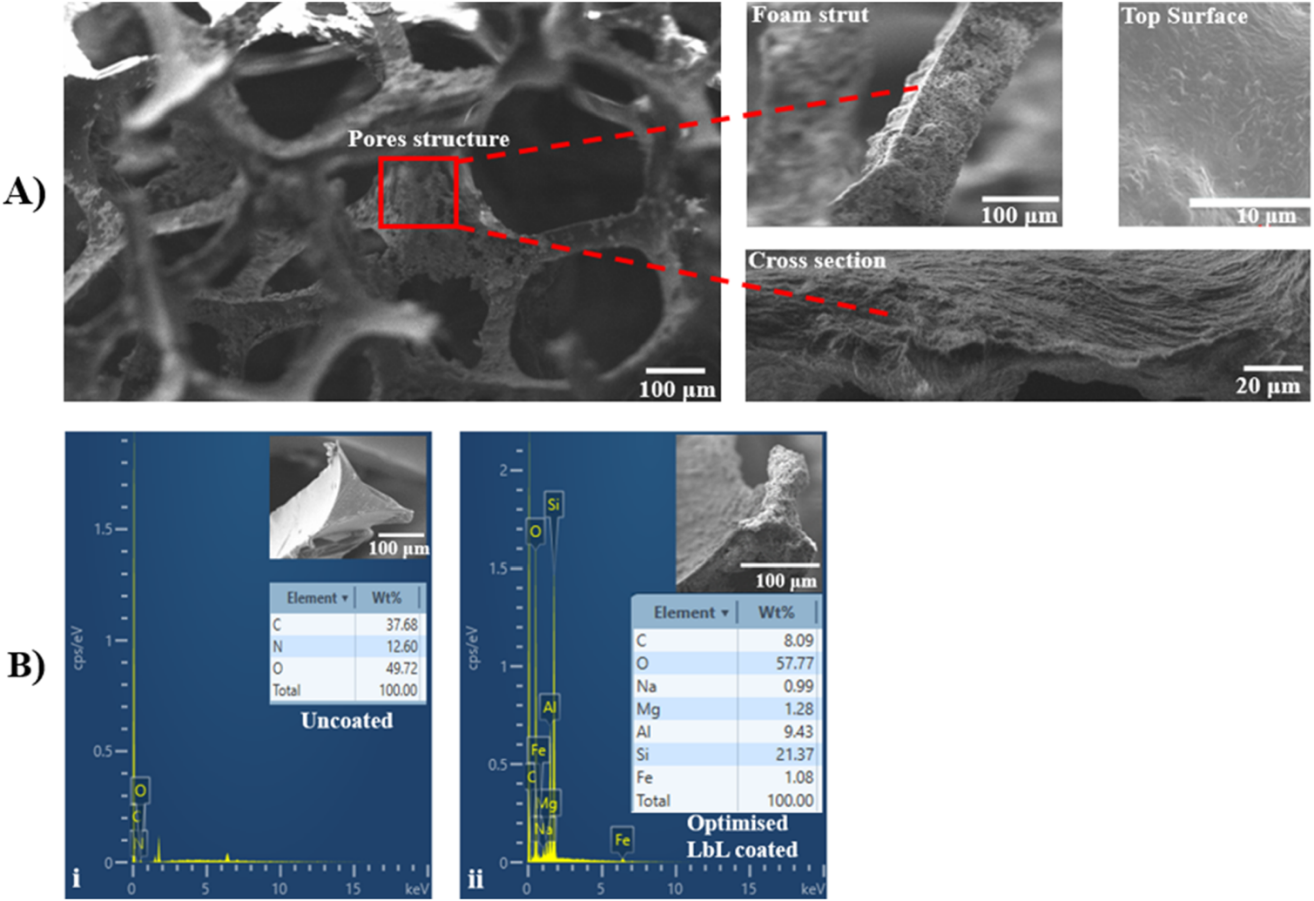
(A) SEM analysis confirmed the successful LbL deposition of a multilayer coating into the interconnected structure, covering uniform pore surfaces and the struts of the scaffold under optimised conditions. (B) EDX analysis, (i) uncoated scaffold, and (ii) investigation of the elemental composition of the optimised parameters LbL-coated scaffold and corresponding tables with the SEM images.

SEM and EDX analyses were performed on optimised coated scaffolds. SEM images demonstrated uniform, dense, and homogeneous multilayer coatings within the interconnected scaffold structure and on the surface, with increased coating thickness observed at 100 QLs (Fig. 9a). EDX analysis confirmed the successful incorporation of nanoclay into the polymer systems, forming a nanocomposite coating. The EDX spectra (Fig. 9b) showed characteristic elemental peaks for aluminium (9.43%) and silica (21.37%), consistent with the chemical composition of MTM nanoclay, specifically (Na, Ca)0.33(AlMg)_2_(Si_4_O_10_)(OH)_2_.

#### 3.3.2 Mechanical properties

**Fig. 10:**
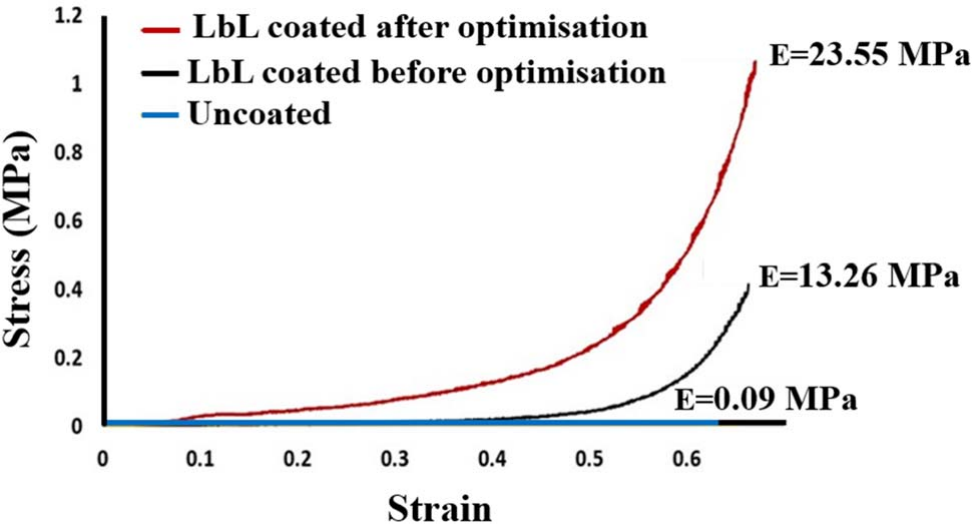
Strain–stress graph of the uncoated scaffold, LbL-coated before the optimisation process, and LbL-assembled coated under optimised conditions (100 QLs deposited).

The mechanical performance of the PU scaffolds was directly influenced by the LbL assembly parameters. Uncoated scaffolds (45 PPI) exhibited a low elastic modulus of 0.09 MPa, consistent with the highly porous structure of the base PU foam. LbL deposition progressively increased stiffness as a function of layer number and deposition conditions. Scaffolds coated under non-optimised conditions reached an elastic modulus of 13.26 MPa, indicating partial reinforcement due to incomplete coating coverage and limited nanoclay integration. Under optimised deposition parameters, the elastic modulus further increased to 23.55 MPa (Fig. 10), reflecting the formation of a continuous and well-integrated nanocomposite layer along the foam struts. The optimised LbL process thus produced a mechanically cohesive hybrid scaffold with substantially enhanced modulus and improved load-bearing capacity.

#### 3.3.3 Porosity

**Fig. 11:**
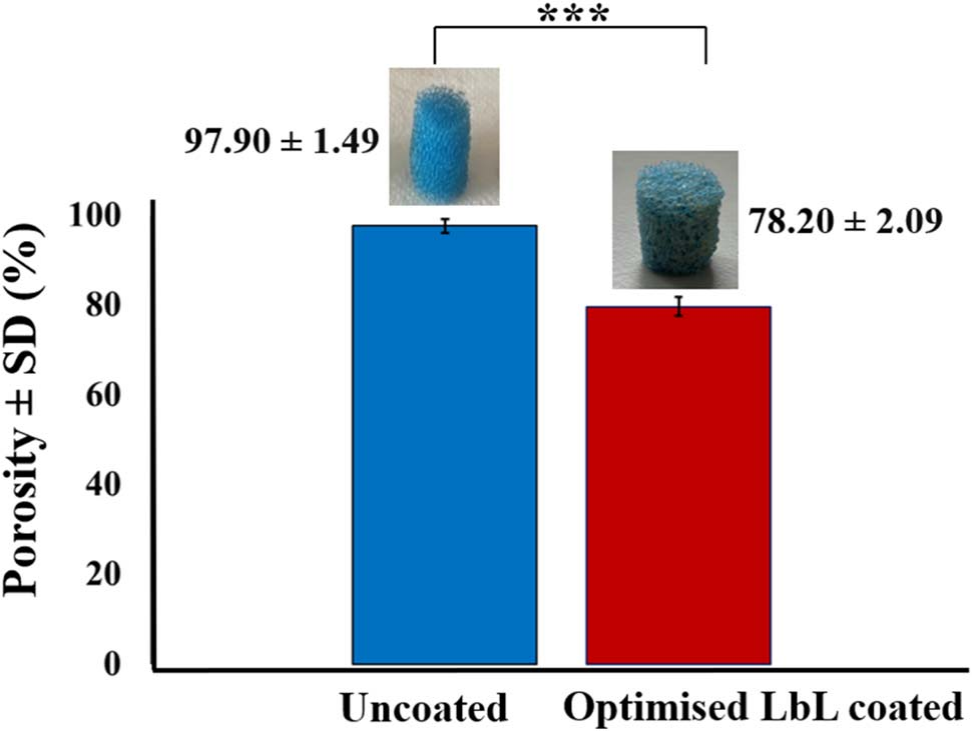
Porosity test results of the uncoated scaffold and LbL-coated under optimised conditions. The values are mean ± standard deviation (*n* = 5), ***p-value < 0.001.

Porosity analysis demonstrated that LbL coating significantly modified the scaffold architecture. The optimised LbL-coated scaffolds (100 QLs) showed an 18.70% reduction in porosity, decreasing from 97.90% ± 1.49 in the uncoated scaffolds to 78.20% ± 2.09 (*p*-value < 0.001, Fig. 11). Despite this reduction, the coated scaffolds maintained a well-interconnected pore network suitable for bone tissue ingrowth while exhibiting enhanced mechanical strength. These findings highlight the role of controlled LbL deposition in fine-tuning scaffold architecture to balance porosity and mechanical performance. The initially high porosity of the uncoated scaffolds facilitated the application of multiple coating layers without reducing porosity below the minimum threshold generally regarded as necessary for bone tissue engineering (>60%).^30^

#### 3.3.4 Chemical composition of coating

**Fig. 12:**
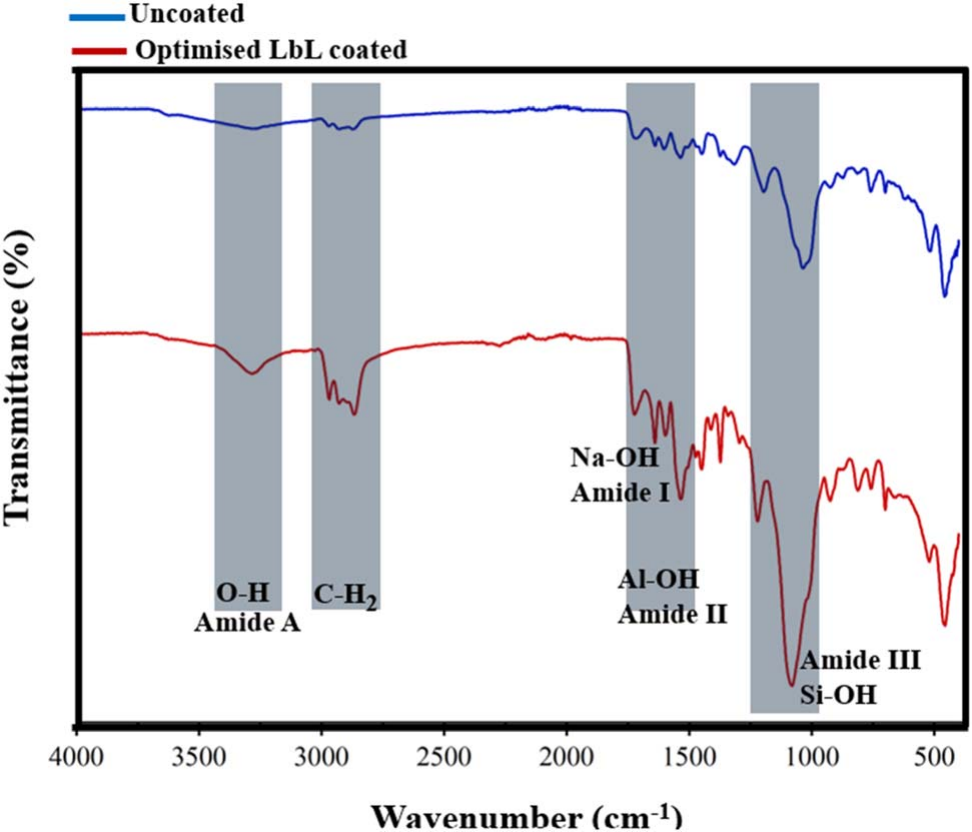
FTIR spectra of the uncoated scaffold and LbL-coated scaffold prepared under optimised conditions.

FTIR and XRD analyses were performed on LbL-coated scaffolds fabricated under optimised conditions, confirming the presence of nanocomposite coatings on the scaffold. FTIR spectra exhibited characteristic absorption bands corresponding to organic and inorganic components reported in the literature. Specifically, five prominent peaks indicated nanoclay incorporation: amide A of biopolymers (PLL and PGA) and the hydroxyl group of nanoclay at 3276 cm^−1^; CH_2_ of PLL and PGA proteins at 2973 cm^−1^; amide I and sodium hydroxide groups at 1645 cm^−1^; amide II and aluminium hydroxide groups at 1542 cm^−1^; and amide III and silicon hydroxide groups at 1073 cm^−1^ (Fig. 12).^16^

**Fig. 13:**
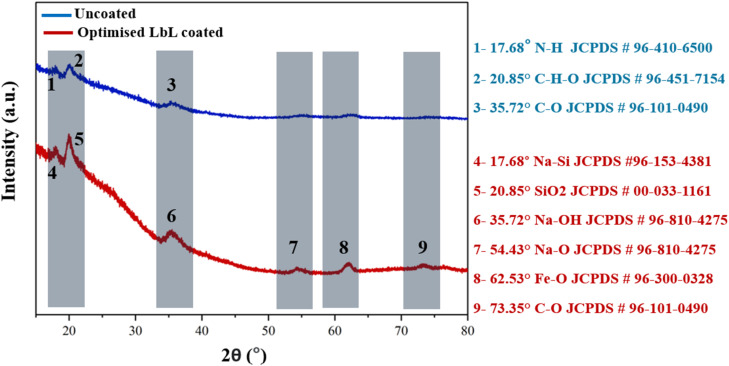
Representative XRD spectra of the uncoated scaffold and the LbL-coated scaffold prepared under optimised conditions.

XRD analysis served as a secondary qualitative technique to verify the presence of nanoclay in the coating. Refinement of XRD data confirmed the incorporation of nanoclay, consistent with FTIR results. Fig. 13 presents representative XRD patterns for multilayer PLL/PGA/PDDA/nanoclay coatings alongside the uncoated scaffold. Coated scaffolds exhibited six distinct peaks at approximately 17.68°, 20.85°, 35.72°, 54.43°, 62.53°, and 73.35°, characteristic of MTM nanoclay.^49^ These peaks correspond to Na–Si (JCPDS #96-153-4381), SiO_2_ (JCPDS #00-033-1161), Na–OH (JCPDS #96-810-4275), Na–O (JCPDS #96-810-4275), Fe–O (JCPDS #96-300-0328), and C–O (JCPDS #96-101-0490), respectively.^50^ Low-angle peaks at 17.68° (N–H, JCPDS #96-410-6500), 20.85° (C–H–O, JCPDS #96-451-7154), and 35.72° (C–O, JCPDS #96-101-0490) were also present in the uncoated scaffold, indicating that the LbL process did not alter the scaffold's fundamental structure.^51^ The observed increase in gallery spacing suggests intercalation of PLL and PGA into the nanoclay during deposition. Similar increases in *d*-spacing have been reported for LbL assemblies of graphene oxide/poly(vinyl alcohol) (from 8.3 Å to 13.2 Å) and PEI/PAA/LPEI/nanoclay films (from 11.4 Å for pure MTM to 13.3 Å for PEI/PAA/LPEI/nanoclay QLs).^52,53^ The smaller *d*-spacing increase observed here may result from differences between the spectra of pure MTM and MTM-Na^+^ (cloisite Na^+^) nanoclay. Overall, XRD results confirm the successful incorporation of nanoclay into LbL coatings, contributing to improved physico-mechanical properties of the scaffolds.

#### 3.3.5 Wettability of coating

**Fig. 14:**
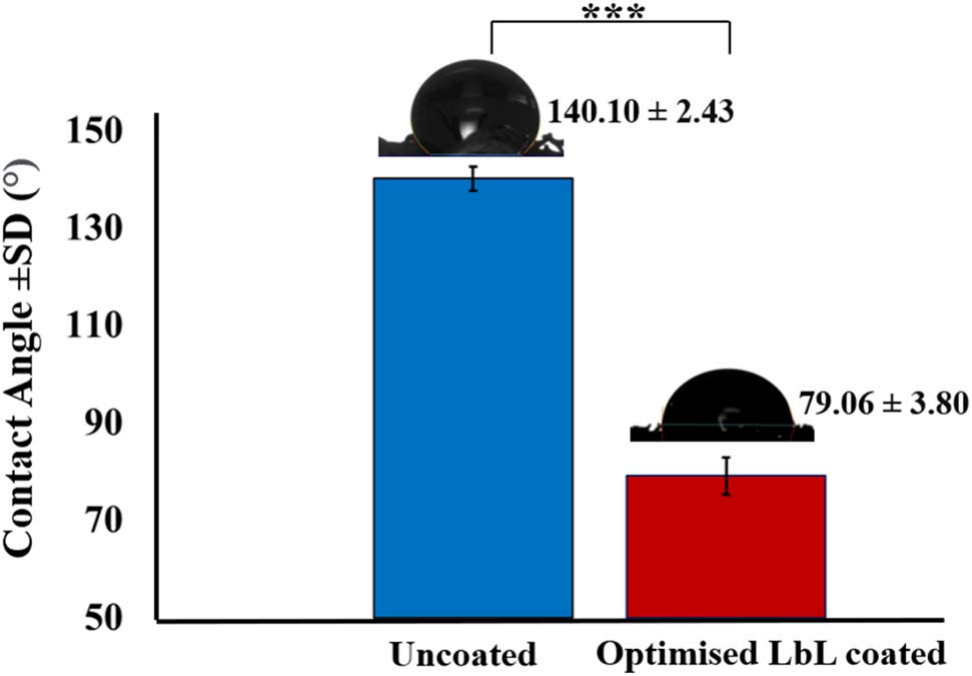
Contact angle measurement of the uncoated scaffold compared to the LbL-coated scaffold prepared under the optimised conditions. The values are presented as the mean ± standard deviation (*n* = 5). ***p-value < 0.001.

Water contact angle measurements were performed to assess the surface wettability of the scaffolds. The LbL-coated scaffold produced under optimised conditions exhibited a significant reduction in contact angle to 61.04° ± 1.37 (*p*-value < 0.001) compared with the uncoated scaffold (Fig. 14), indicating increased surface hydrophilicity. This enhanced hydrophilicity is attributed to the exposure of hydroxyl (–OH) functional groups from the polyelectrolyte layers within the LbL coating. For 3D porous scaffolds, improved wettability is particularly advantageous, as it promotes fluid infiltration and enhances interfacial interactions across the scaffold architecture. The wettability achieved through the optimised LbL process aligns with previously reported surface characteristics known to support favourable biological responses in bone tissue engineering applications.^54^

## 4 Discussion

Many scaffold materials exhibit high porosity, which limits load-bearing capacity and reduces the ability to tailor mechanical properties to specific requirements.^55,56^ The LbL assembly technique offers a method to reinforce porous scaffolds with nanostructured coatings while preserving their interconnected architecture.^9^ Mechanical and physical properties of cellular materials, such as open-cell scaffolds, depend strongly on their base material, relative density, and pore morphology.^26^ Controlling these properties is important for applications including engineered bone scaffolds, high-stiffness lightweight structures, and advanced packaging.^51^ Incorporating stiff nano- and micro-composite coatings through LbL assembly into 3D porous cellular structures provides a feasible reinforcement strategy.^9,21^ Nonetheless, comprehensive parametric studies optimising this assembly process remain limited.^9,16^

While the application of LbL coatings to porous PU foam scaffolds has been reported previously,^16,30,31,46^ the present study advances prior work by introducing a structured DoE-driven optimisation approach applied to a distinct nanocomposite material system comprising PLL/PGA/PDDA/MTM. In contrast to earlier studies, two independent DoE analyses were performed to evaluate composition- and process-related factors separately, enabling identification of critical variables and interactions governing multilayer formation and mechanical reinforcement. Beyond parameter optimisation, this work provides insight into how polymer/nanoclay concentration, solution pH, and LbL processing parameters collectively influence electrostatic interactions, nanoclay dispersion, coating morphology, interfacial bonding, and the resulting physicochemical and mechanical properties of the multilayer-coated scaffolds. Moreover, optimised and non-optimised scaffolds were systematically compared using mechanical and physicochemical characterisation, facilitating a clearer understanding of structure–property relationships in 3D highly porous systems. Two DoE studies identified key factors affecting scaffold properties, including coating thickness, mass, porosity, and compressive elastic modulus. DoE study 1 focused on material factors such as polymer and nanoclay pH and concentration, while DoE study 2 evaluated processing parameters including pore density, coating time, number of layers, and drying frequency. Statistical analyses, including half-normal probability plots and ANOVA, were used to determine the main contributors to each property. Optimisation aimed to maximise compressive modulus while maintaining high porosity and minimising coating thickness and mass. Under optimised conditions, significant improvements were achieved in tailoring both surface and bulk scaffold properties through nanocomposite LbL coatings. As the LbL assembly technique relies on electrostatic interactions between oppositely charged materials, solution pH critically influences coating behaviour.^57^ DoE study 1 aimed to optimise the concentration and pH parameters of the nanocomposite coating material system. The optimal conditions were 1 wt% for polymers and 0.5 wt% for nanoclay, with pH values selected to maximise electrostatic polyelectrolyte interactions based on charge considerations (PLL pH 3.5, PGA pH 10, PDDA pH 4.5, and MTM pH 9). Variations in pH modulate the ionisation of functional groups within the polyelectrolytes, altering charge density and thereby affecting attraction strength and film growth kinetics.^58^ Under the optimised conditions identified by DoE study 1, the balance of polymer and nanoclay concentrations with pH maximised electrostatic binding, producing more cohesive and mechanically robust coatings. It was hypothesised that low pH for polycations such as PLL and PDDA, combined with high pH for the negatively charged MTM nanoclay and polyanion PGA, enhances binding interactions within the LbL coating. These conditions improve electrostatic attraction, facilitating stronger and more uniform coatings since electrostatics predominates the multilayer film assembly.^58,59^ The enhanced physicochemical and mechanical properties observed at lower polymer and MTM nanoclay concentrations can be attributed to improved particle diffusion and uniform layer deposition within the 3D porous scaffold. At higher concentrations, restricted diffusion and particle aggregation reduce coating homogeneity, leading to diminished mechanical performance.^57,60^ Additionally, increasing concentration reduces scaffold porosity as pores become filled, limiting internal surface area available for cellular functions.^16^ MTM concentration and pH significantly influenced all measured scaffold properties. Specifically, variations in MTM levels affected coating thickness, porosity, and mechanical strength.^61,62^ In this study, adjusting MTM pH-controlled nanoclay particle charge density and dispersion, and a higher pH improved particle dispersion and produced more uniform layers, contributing to increased compressive elastic modulus. These observations suggest an optimal balance of concentration and pH was essential for tailoring scaffold properties suited to bone tissue engineering applications.^9^ These findings align with previous studies using MTM nanoclay LbL coatings to enhance PU scaffold physical and mechanical characteristics.^31,46^ Scaffold structure and physical properties exhibited predictable trends: strut thickness and mass increased while pore size and porosity decreased with thicker coatings, consistent with observations by Ziminska *et al.*^30^ The optimised scaffold from DoE study 1 displayed over 100% improvement in mechanical properties compared to the pre-optimised LbL-coated scaffold, demonstrating the effectiveness of the identified coating parameters. Collectively, the optimisation of pH and component concentration enables controlled manipulation of charge density, nanoclay dispersion, and polymer-nanoclay interfacial interactions, governing multilayer formation and the resulting structural reinforcement.

DoE study 2 conducted a parametric investigation of LbL coating parameters, identifying optimal conditions of a scaffold pore density of 45 PPI, 100 QLs, a coating time of 30 s, and drying every 10 QLs. Increasing coating time and the number of layers resulted in greater coating thickness, likely due to enhanced diffusion, consistent with previous studies on clay-based thin films.^63^ Scaffold pore density and the number of deposited QLs were the primary parameters affecting scaffold properties. In DoE study 2, optimisation analysis indicated that scaffold pore density influences coating performance by altering accessible surface area and internal diffusion pathways within the 3D architecture. Increasing the number of deposited QLs promoted progressive LbL nanocomposite build-up, thereby enhancing interfacial coverage and further improving the scaffold's physico-mechanical properties. This study presents the first parametric analysis demonstrating how pore density and layer number influence the physico-mechanical properties of polymer-based scaffolds during LbL coating. The combination of 45 PPI and 100 QLs, alongside optimised drying and coating times, yielded a 260-fold increase in mechanical properties, emphasising the capacity of the LbL process to enhance scaffold mechanics. Similarly, Acheson *et al.* reported an increase in elastic modulus from 0.08 ± 0.01 MPa for uncoated PU scaffolds to 4.90 ± 0.46 MPa for PEI/PAA/PEI/nanoclay coatings with 60 QLs.^46^ They observed that mechanical properties rose with the number of QLs, highlighting the importance of optimising layer count in LbL fabrication for bone tissue engineering. The drying interval of 10 QLs and 30 s dipping time align with previous findings by Ziminska *et al.*, McFerran *et al.*, and Acheson *et al.*, who reported improvements in mechanical strength when applying drying post every 10 QLs and using 30 s dip times on PU scaffolds.^16,30,31,46^ This consistency supports the reliability of these processing parameters across studies.

Throughout all experiments, scaffold porosity remained above 60%, meeting requirements for cancellous bone substitutes.^64^ This porosity enabled deposition of additional coating layers to tailor mechanical properties without compromising minimum porosity targets.^16^ Optimised LbL-coated scaffolds preserved high porosity and an interconnected pore network essential for cell infiltration.^23^ The scaffolds exhibited a mass increase of 2.41 ± 0.55 mg and coating thickness of 1.86 ± 0.43 µm per QL, proportional to layer number. SEM confirmed uniform nanocomposite coatings, with pore size reduced from 816.4 ± 9.2 µm (uncoated) to 424.1 ± 12.1 µm (LbL-coated). A significant improvement in mechanical properties from 0.082 to 21.64 MPa was achieved while maintaining 78.2 ± 1.24% porosity. McFerran *et al.* similarly demonstrated increases from 0.15 ± 0.10 MPa (uncoated) to 6.01 ± 0.36 MPa for a PU-based scaffold with PDDA/PAA/PDDA/nanoclay coatings of 60 QLs.^31^ These findings highlight the necessity of controlling LbL assembly parameters precisely to achieve the desired mechanical performance in porous scaffolds for biomedical applications.

The DoE studies demonstrated that the compressive elastic modulus of LbL-coated scaffolds was influenced either by the amount of deposited coating material, which increased coating thickness and mass, or by changes in assembly conditions that modified the coating's mechanical properties. The LbL-deposited MTM nanoclay exhibits a high aspect ratio, strong intrinsic stiffness, and marked electrostatic affinity for polyelectrolytes (DoE 1), making it well suited for mechanical reinforcement in nanocomposite coatings. Optimisation of LbL processing parameters (DoE 2) enhanced electrostatic attraction between MTM platelets and oppositely charged polyelectrolyte layers, producing thinner, denser, and more structurally stable multilayers. Improved particle mobility during deposition promoted uniform MTM distribution and minimised aggregation, yielding a more homogeneous coating with increased density. These features strengthened interlayer cohesion and adhesion between the coating and the scaffold substrate. Consequently, the LbL nanocomposite coating functioned as a mechanically integrated shell on the scaffold struts, where well-dispersed MTM platelets enabled efficient load transfer and robust interfacial bonding. This reinforcement mechanism accounts for the ∼260-fold increase in compressive modulus following optimisation. The LbL nanocomposite coating fabricated under optimised conditions exhibited a more homogeneous and cohesive morphology, as shown by SEM (Fig. 9A), indicating improved dispersion of MTM platelets and enhanced coating integrity. Optimisation of the LbL assembly parameters improved electrostatic interactions between MTM nanoclay platelets and the oppositely charged polyelectrolyte layers, leading to tighter interlayer packing and improved mechanical cohesion within the multilayered film. Complementary chemical analyses supported these morphological observations. ATR-FTIR spectra of scaffolds prepared under the optimised DoE parameters showed greater intensities for hydroxyl, methylene, and amide functional bands, consistent with stronger intermolecular interactions and increased structural stability of the LbL architecture (Fig. 12). Furthermore, EDX mapping and XRD analysis confirmed uniform MTM distribution and partial intercalation within the polyelectrolyte matrix (Fig. 9B and 14), both contributing to improved load transfer across the multilayer structure. Surface analysis of optimal LbL-coated scaffolds showed increased hydrophilicity, evidenced by a 33% decrease in contact angle from 139.57 ± 1.86° to 93.48 ± 2.14°. This improvement in wettability can enhance cellular adhesion and spreading, key factors in bone scaffold performance.^65,66^ Collectively, these results demonstrate that the optimised LbL conditions identified through DoE facilitated controlled nanoclay dispersion and reinforced interlayer bonding, which directly underpins the enhanced mechanical and physicochemical performance of the coated scaffolds. Our optimisation confirmed the capability to tailor surface and bulk properties of highly porous scaffolds through LbL assembly, aligning with recent findings by Ziminska *et al.* (2019), McFerran *et al.* (2022), Acheson *et al.* (2023), and Sahebalzamani *et al.* (2024), who emphasised the potential of controlling LbL assembly parameters to modulate scaffold physico-mechanical characteristics for biomedical applications.^16,30,31,46^ Increasing the number of QLs or introducing crosslinkers could further improve stiffness and strength to approximate the compressive modulus of cancellous bone. The mechanical properties of the LbL-coated scaffolds reflected the influence of coating characteristics arising from different assembly conditions.^9,10^ A DoE-based optimisation approach was used to balance the trade-off between mechanical reinforcement and porosity retention – both critical parameters for bone scaffold design. Scaffolds produced under optimised conditions exhibited a marked increase in elastic modulus from 0.09 MPa to 23.55 MPa while maintaining high porosity (78.2 ± 1.24%). Although these values remain below the typical range reported for cancellous bone (elastic modulus: 0.05–0.5 GPa), the optimised scaffolds demonstrate mechanical suitability for non-load-bearing bone tissue engineering applications.^55,56^ Furthermore, the retained porosity provides adequate internal volume for additional QL deposition, allowing further mechanical adjustment if required. Surface characterisation revealed that optimised LbL coatings substantially increased hydrophilicity, which may enhance cell–material interactions and support cellular attachment and matrix integration during the early stages of repair and regeneration applications.^54^ Comprehensive biological evaluation, including assessment of cytocompatibility, and the ability to support bone cell adhesion, proliferation, and differentiation, remains essential to fully validate functional performance. These investigations will form the focus of future work aimed at confirming the biological efficacy of the optimised scaffold platform.

This study is, to our knowledge, the first to simultaneously optimise material system parameters (concentration and pH) and process parameters (pore density, number of layers, coating time, and drying cycles), resulting in significant enhancement of mechanical and chemical scaffold properties, confirming that LbL assembly can produce thin, uniform films within porous structures suitable for bone tissue engineering. Future work should focus on determining the minimal number of QLs to replicate cancellous bone mechanical properties and exploring alternative crosslinking strategies to maintain mechanical integrity under hydrated physiological conditions. Furthermore, *in vitro* studies on optimised scaffolds are required to evaluate cell adhesion and proliferation and establish their suitability for *in vivo* bone tissue engineering applications.

## 5 Conclusions

Multilayered films fabricated using LbL assembly enabled precise control over coating thickness, composition, and morphology by adjusting assembly parameters such as the number of layers, solution pH, constituent composition, rinsing times, intermittent drying, and coating duration. This study systematically evaluated the effects of LbL assembly parameters and coating material systems on open-cell porous polymer-based scaffolds coated with PLL/PGA/PDDA/MTM nanoclay composites using two DoE studies focused on materials and process parameters. Optimisation of the polymer-nanocomposite coatings through LbL assembly demonstrated that the surface and bulk properties of LbL-coated porous scaffold can be tailored to meet specific application requirements. This study identifies the dominant parameters governing multilayer formation in LbL nanocomposite coatings and elucidates how pH and concentration-dependent electrostatic interactions determine coating architecture and the corresponding physicochemical and mechanical performance of porous scaffolds. The findings showed that sufficient layer deposition of nanoclay-reinforced coatings produced mechanical properties comparable to those required for bone tissue scaffolds. Crucially, the optimal pH and material system conditions were defined based on achieving high electrical charge densities. Process parameters, including pore density, layer number, coating time, and drying steps, were also optimised. The methodology established provides a reproducible approach for tailoring scaffold properties for bone tissue engineering. This comprehensive optimisation framework facilitates the fabrication of polymer-nanocomposite coatings on highly porous scaffolds with controlled mechanical and physical properties, porosity, and functional incorporation.

## Conflicts of interest

There are no conflicts to declare.

## Data Availability

The data supporting the findings of this study are included within the article. Additional raw data can be made available by the corresponding author upon reasonable request that ensure data protection and appropriate use.
